# Association Between Total Cholesterol and In-Hospital Mortality Among First-Episode Acute Coronary Syndrome Patients: Multilevel Analysis from NCVD-ACS Registry

**DOI:** 10.3390/healthcare14162620

**Published:** 2026-08-19

**Authors:** Tg Mohd Ikhwan Tg Abu Bakar Sidik, Shamsul Azhar Shah, Sazzli Kasim, Gunavathy Selvaraj, Kien Ting Liu, Nazarudin Safian, Wan Azman Wan Ahmad

**Affiliations:** 1 Department of Public Health Medicine, Faculty of Medicine, Universiti Kebangsaan Malaysia, Kuala Lumpur 56000, Malaysia; tgikhwancrc@gmail.com (T.M.I.T.A.B.S.); nazarudin@ppukm.ukm.edu.my (N.S.); 2 Cardiovascular Advancement and Research Excellence Institute (CARE Institute), Universiti Teknologi MARA, Sungai Buloh 47000, Malaysia; sazzlikasim@gmail.com; 3 National Heart Association of Malaysia, Kuala Lumpur 59200, Malaysia; gunavathy@malaysianheart.org (G.S.); kienting@malaysianheart.org (K.T.L.); 4 Department of Medicine, University Malaya Medical Centre, Kuala Lumpur 59100, Malaysia; wanazman@ummc.edu.my

**Keywords:** heart attack, hierarchical models, cholesterol paradox, in-hospital mortality

## Abstract

**Background:** Cardiovascular disease (CVD) remains the leading cause of mortality worldwide, particularly in low- and middle-income countries. Although elevated cholesterol is a well-established modifiable risk factor for atherosclerotic CVD, the phenomenon known as the “cholesterol paradox” has raised uncertainty regarding the prognostic significance of admission cholesterol levels in patients with acute coronary syndrome (ACS). Therefore, this study aimed to evaluate the association between admission total cholesterol (TC) levels and in-hospital mortality among patients with first-episode ACS. **Methods:** Data were obtained from the National Cardiovascular Disease Database-Acute Coronary Syndrome (NCVD-ACS) registry, where this cross-sectional study implemented a consecutive sampling of the nationwide ACS patient database for eligible records from January 2006 to December 2015. The patients were then classified into quartiles according to their TC levels, with the corresponding all-cause in-hospital mortality considered the outcome. Following adjustments for potential confounding factors, multilevel analysis ensued to determine the association between cholesterol groups and mortality. **Results:** The quartile two (Q2) group (4.50–5.36 mmol/L) was employed as the reference for this study. From the 16,860 ACS cases (mean age = 54.1 years; 83.9% male) analysed, which included 648 in-hospital fatalities, lower cholesterol levels were linked to higher mortality rates, except among patients with unstable angina. Interestingly, patients with non-ST-segment elevation myocardial infarction (NSTEMI) demonstrated a U-shaped trend. The results also revealed adjusted odds ratio (OR) for mortality (95% CI: 1.15–4.03) for Q1 at 2.15 (2.00–4.49 mmol/L), 2.01 (95% CI: 1.01–3.98) for Q3 (5.37–6.25 mmol/L), and 2.15 (95% CI: 1.08–4.26) for Q4 (>6.25 mmol/L), suggesting an increased mortality among patients with low and high cholesterol levels. **Conclusions:** Among first-episode ACS patients, both low and high cholesterol levels were associated with increased in-hospital mortality. A significant U-shaped relationship was observed among NSTEMI patients.

## 1. Introduction

Although research in cardiovascular risk factors has been ongoing for decades, the association between cholesterol and mortality, particularly in acute coronary syndrome (ACS) patients, is still not completely comprehended. The World Health Organisation (2020) [1] revealed that approximately a third of mortalities worldwide were due to non-communicable diseases, among which cardiovascular diseases (CVD) are the most significant cause. Ischemic heart disease and stroke also led to about 25% of all mortality, making them the leading causes of fatalities.

Hypertension, diabetes, high cholesterol levels, obesity, and smoking are among established and modifiable risk factors that are predominant contributors to developing CVD [2]. In Malaysia, significant cholesterol levels and obesity are more prevalent than the other risk factors, recording 38.1% and 50.1%, respectively. Rising almost twofold from 20.7% (2006) to 38.1% (2019), substantial cholesterol levels have been considerably increasing, while obesity remained unaltered despite its substantial prevalence.

High cholesterol, which includes hypercholesterolemia or hyperlipidaemia, refers to the notable levels of cholesterol in the blood. Although an essential lipid required for normal physiological functions, excessive cholesterol can contribute to the development of various health issues [3]. Elevated lipids, primarily low-density lipoprotein cholesterol (LDL-C), are also a key risk factor for developing coronary heart disease and atherosclerosis [2]. Furthermore, the Framingham Risk Score suggested that cholesterol can contribute up to 5% to the risk of CVD [4]. Therefore, managing cholesterol is one of the most effective methods to prevent CVD as a modifiable risk factor. The strategy can also reduce CVD incidence, therefore diminishing mortality rates [5].

Cholesterol is necessary for a normal human body to function properly. Significantly low cholesterol levels may compromise the ability of the body to ward off infections and diseases [6]. Low cholesterol levels can arise from certain conditions, such as malnutrition or chronic liver disease, which also increase mortality risk [7,8]. Meanwhile, elevated lipid levels are generally associated with an increased risk of death in a putative causal relationship. This hypothesis was postulated from the understanding that atherosclerosis and coronary artery disease (CAD) arise from substantial cholesterol levels, which then can result in CVD and mortality.

Reports regarding cholesterol and all-cause mortality in CVD patients have demonstrated inconsistencies, giving rise to the cholesterol paradox, which challenges the traditional view that low cholesterol levels are always good. Several studies observed increased mortality among patients with lower cholesterol levels. This observation indicated an inverse association between the parameters [9,10,11]. Meanwhile, other reports recorded a J- or U-shaped relationship. These reports revealed that individuals with low and high cholesterol levels had an elevated mortality risk, while the lowest risk was associated with those with moderate or normal levels [12,13,14]. Consequently, clarifying the relationship between cholesterol and mortality in future research should include varying clinical settings and populations.

CAD, one of the most common manifestations of CVD, arises from progressive atherosclerotic narrowing of the coronary arteries. Acute coronary syndrome (ACS) represents the acute clinical presentation of CAD, most commonly resulting from rupture or erosion of an atherosclerotic plaque with superimposed coronary thrombosis and sudden interruption of coronary blood flow. ACS encompasses a spectrum of conditions, including ST-elevation myocardial infarction (STEMI), non-ST-elevation myocardial infarction (NSTEMI), and unstable angina (UA), distinguished by the extent of myocardial ischaemia and the resulting electrocardiographic and biomarker findings. Despite advances in reperfusion therapy, antithrombotic treatment, and secondary prevention, ACS remains associated with substantial short-term mortality.

Given this continued burden, identifying readily available prognostic markers that could improve early risk stratification in ACS remains an important clinical priority. This study assessed data from Malaysian patients with first-episode ACS to examine the association between admission cholesterol levels and in-hospital mortality. The findings could help clarify the contradictory evidence from previous studies regarding how cholesterol levels influence mortality risk in this population. This study contributes to the existing literature in several respects: it draws on one of the largest nationwide ACS registries in Southeast Asia, a region underrepresented in prior cholesterol paradox research; restricts analysis to first-episode ACS to reduce bias from recurrent events; applies multilevel modelling to account for clustering effects; and examines heterogeneity in this association across ACS subtypes, including STEMI, NSTEMI, and UA.

## 2. Materials and Methods

### 2.1. Study Settings and Design

The Malaysian National Cardio-vascular Database (NCVD-ACS) registry offers a multicentre, observational study. This study analysed NCVD-ACS data from patients registered between January 2006 and December 2015, corresponding to the most recent fully cleaned, validated, and outcome-verified dataset available at the time the study protocol and ethics application were developed. Restricting the analysis to this predefined dataset ensured complete mortality ascertainment and consistent data collection across all participating data providers throughout the study period. This study included ACS patients from 16 data providers (SDP) or state hospitals in Malaysia, which included 11 tertiary hospitals with cardiology departments, three tertiary hospitals without a cardiology unit, one academic teaching hospital, and the National Heart Institute. This comprehensive database is supported by the Malaysian Ministry of Health and the National Heart Association of Malaysia and provides a wide range of information on ACS patients. Therefore, the anonymised patient data employed in this cross-sectional study were sourced from the registry, with clinical profiles, management, and outcomes extracted. Patient-informed consent was waived for this study, considering that the Malaysian Medical Review and Ethics Committee approved the NCVD-ACS registry (approval code: NMRR-07-20-250). Detailed descriptions of the registry were discussed in a prior report [15].

### 2.2. Population and Sampling

This study was centred on Malaysian citizens who had experienced an ACS event, with the NCVD-ACS registry as the sampling frame. As the largest ACS database in the country [16], the registry provides a comprehensive and representative sample of ACS patients in the nation. The consecutive sampling of patients implemented in this study of individuals meeting the inclusion and exclusion criteria ensured a representative of the target population, and minimised selection bias [17].

Malaysians between 30 and 70 years old diagnosed with ACS for the first time were considered as candidates for this study. This age range was pre-specified in the study protocol to represent the age group most commonly affected by typical atherosclerotic ACS in Malaysia [18] and to reduce clinical heterogeneity and confounding. Patients younger than 30 years were excluded as they represented a very small proportion of the registry cohort and may present with ACS through distinct, non-atherosclerotic mechanisms atypical of the broader ACS population. Patients older than 70 years were excluded because of their disproportionately higher burden of comorbidities, frailty, and competing causes of mortality, which could independently influence both cholesterol levels and short-term survival, thereby confounding the association of primary interest. Meanwhile, ACS patients without a measured lipid profile within 48 h of admission and those with a previous history of heart disease (myocardial infarction, documented coronary artery disease, and heart failure) or chronic illnesses (chronic renal and chronic lung diseases) were excluded. These parameters allowed this study to control for potential confounding factors that might affect the association between cholesterol levels and mortality.

### 2.3. Sample Size and Sampling Procedures

**Figure 1:**
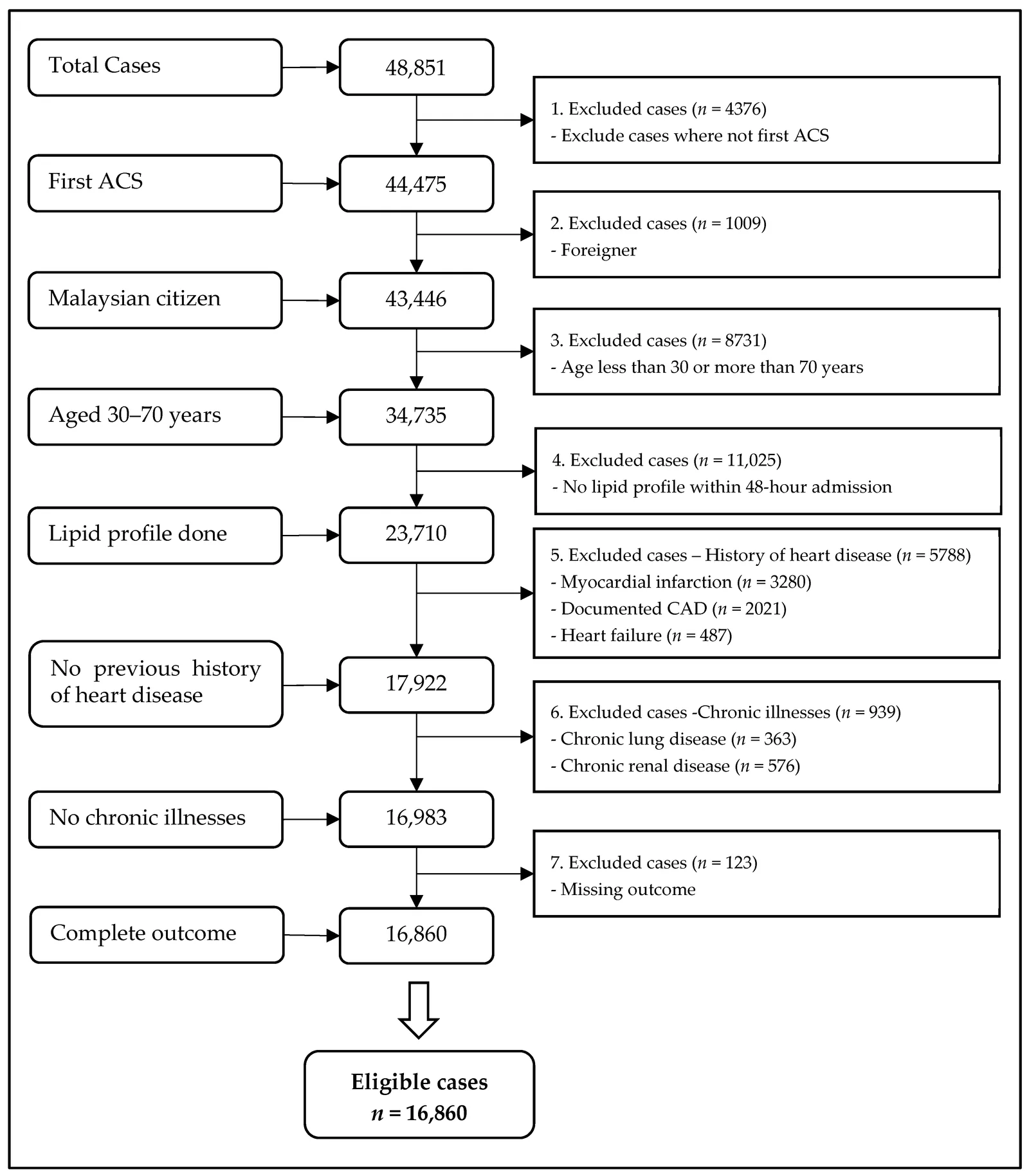
The flowchart for selecting cases from NCVD-ACS to define the study sample (*n* = 16,860 patients).

Between 2006 and 2015, 48,851 cases were recorded by the NCVD-ACS registry. A seven-step case identification process to determine study participants is applied in this study, as illustrated in Figure 1. First, 4376 non-initial ACS cases and duplicate records were excluded. Cases involving foreign nationality (1009) and patients younger than 30 years old or older than 70 years old (8731) were also discarded. Subsequently, 11,025 cases were excluded due to the unavailability of lipid profile data within 48 h of admission. In the fifth step, this study discarded cases with a history of heart disease, including myocardial infarction (3280 cases), documented coronary artery disease (2021 cases), and heart failure (487 cases). Furthermore, cases involving chronic ailments, such as chronic lung and renal diseases, were excluded, which accounted for 939 cases. Finally, missing outcome data led to another 123 records being discarded. Consequently, 16,860 cases were analysed in this study.

### 2.4. Study Variables and Outcomes of Interest

The NCVD-ACS registry collects data on patients admitted to hospitals with ACS with a standardised case reporting form (CRF), ensuring data with consistency and quality are obtained across different SDPs in Malaysia. A wide range of information is encompassed in this registry, including demographic information, risk factors, comorbidities, baseline investigation and electrocardiography (ECG) results, clinical diagnosis at admission, in-hospital treatment, pharmacologic therapy, and clinical outcomes. This study modelled the data collection procedure per international registries and guidelines, including those recommended by the European Cardiology Audit and Registration Data Standards, the Australian National Data Elements for ACS, and the American College of Cardiology Clinical Data Standards [15].

The data extracted from the NCVD-ACS registry were categorised into baseline and treatment attributes. In the baseline characteristic group, demographic information (age, gender, and ethnicity), risk factors, comorbidities, baseline investigation, and ECG were included. This study considered smoking status, family history of premature CAD, dyslipidaemia, hypertension, and diabetes mellitus as risk factors, whereas cerebrovascular (CVA) and peripheral vascular diseases were classified as comorbidities. Meanwhile, only baseline variables, such as heart rate (HR), systolic blood pressure (SBP), diastolic blood pressure (DBP), and fasting blood glucose (FBG), measured within 48 h of admission, were included.

In-hospital treatment, pharmacological therapy, and clinical diagnosis were deemed as treatment patterns in this study. The treatments prescribed to each patient were based on their ACS stratum: STEMI, NSTEMI, or UA. This study also recorded the Killip class (I to IV) for each case. Killip class was selected as the primary measure of clinical severity at presentation, given its well-established prognostic value and consistent use in ACS risk stratification. Under treatment patterns, in-hospital cardiac catheterisation, percutaneous coronary intervention (PCI), and coronary artery bypass graft (CABG) were included. On the other hand, pharmacological therapy encompassed all medications received by the patients received during hospitalisation, such as antiplatelet agents, anticoagulants, beta-blockers, and statins.

Lipid profiles were measured as part of routine clinical care at each of the participating NCVD hospitals, using the accredited clinical laboratory and standard operating procedures of that centre, rather than a single central laboratory; the resulting values were subsequently extracted from patients’ medical records into the registry. Admission total cholesterol (TC) was the primary exposure variable in this study. TC was selected, rather than LDL-C or non-high-density lipoprotein cholesterol (non-HDL-C), as it is routinely measured directly and recorded with high completeness across all participating hospitals, whereas calculated LDL-C depends on fasting sampling and loses accuracy at high triglyceride concentrations. TC has also been the parameter most commonly used in previous cholesterol paradox studies, allowing direct comparison with the existing literature. To examine whether the findings were specific to TC, supplementary analyses of LDL-C, HDL-C, triglycerides, and non-HDL-C were additionally performed and are presented in Appendix A, Table A3.

Based on TC profiles, the patients were divided into quartiles, where patients in the first 25 centiles were grouped in the first quartile, while those in the 26th to 50th, 51st to 75th, and 76th to 100th centiles were grouped under the Q2, Q3, and Q4, respectively. This categorisation was implemented according to the cholesterol paradox, which postulated a U-shaped relationship between mortality rate and cholesterol levels.

In this study, the Q2 group was employed as the reference group when comparing the odds ratios (OR) of mortality between the quartile groups [13]. Quartile categorisation was selected to explore potential non-linear and U-shaped associations, while Q2 was used as the reference group because its cholesterol range corresponded most closely to the recommended normal range in NCEP-ATP III guidelines [19]. This study also considered hospital mortality from all causes as the primary outcome of interest. Therefore, a cross-check with the National Registration Department was conducted to validate the mortality status of the patients.

### 2.5. Statistical Analysis

A univariable analysis was performed to determine the association between cholesterol groups and study variables (baseline characteristics and treatment pattern) assessed in this study. For categorical variables, the differences between groups Q1, Q2, Q3, and Q4 were also established with the chi-square or Fisher’s exact test (if the expected minimum count was under five). Meanwhile, a one-way ANOVA evaluation was employed to assess normally distributed numerical variables. This study conducted statistical analyses with Stata software [StataCorp. 2017, Stata Statistical Software: Release 15, College Station, TX, USA]. A two-tailed *p*-value of <0.05 was also considered statistically significant.

The factors affecting the cholesterol group and mortality were determined through a multilevel analysis. In the multilevel logistic regression applied, a random intercept was conducted, in which individuals (level 1) were nested within the SDP (level 2). This study also included SDP-level characteristics, including the estimated target population, number of cardiologists, and number of cathlabs, as predictors in the model.

Determining if multilevel modelling was applied to the data set assessed in this study (evidence of clustered or hierarchical data) was calculated with the intraclass correlation coefficient (ICC) and F statistic. The proportion of variance in the outcome represented by the SDP-level attributes was denoted by ICC. The null hypothesis in this study was that there was no variation in the outcome across the SDPs, and was evaluated with the F statistic [20].

The ICC obtained in this study was 0.067 with a 95% confidence interval (CI) of 0.033 to 0.129. This data indicated that 6.7% of the variance in the outcome variables was due to differences between SDPs. Meanwhile, a 40.65 F statistic was recorded, with a *p*-value of under 0.001. This observation suggested a substantial between-SDP variance, rendering multilevel modelling appropriate [21].

Notable factors affecting the cholesterol groups assessed in this study were determined through multinomial logistic regression. All individual-level variables with a *p*-value of less than 0.05 in univariable analysis were then fitted into the multilevel model. The significant factors were also considered confounders and were adjusted during the mortality analysis. Subsequently, the association between the cholesterol groups and mortality status was established with binary logistic regression.

Based on the selection of confounding variables, this study analysed four models. Model 1 was unadjusted and was without confounding variables, while Model 2 was only adjusted for significant baseline characteristics. Treatment pattern variables were incorporated in Model 3, and Model 4 included all variables at both levels through multilevel modelling. The magnitude and direction of the association between the cholesterol group and mortality rate were also assessed using OR with 95% CI values. Subsequently, the goodness of fit of each model was determined according to the Akaike information criterion (AIC), Bayesian information criterion (BIC), and −2 log likelihood (−2LL).

## 3. Results

### 3.1. Demographic Characteristics

In this study, 16,860 ACS cases documented by Malaysian hospitals between 2006 and 2015 were assessed, which included 14,142 (83.9%) and 2718 male and female patients. Among the patients between 30 and 70 years old, their mean age was 54.1 years, with a standard deviation (SD) of 9.0 years. Most patients were also Malay (56.4%), followed by Chinese (19.0%), Indians (19.7%), and others (4.9%).

### 3.2. Cholesterol with Baseline Characteristics and Treatment Patterns

Patients’ baseline characteristics and treatment patterns based on their TC levels are summarised in Table 1 and Table 2. Based on the data, patients with low cholesterol levels were significantly older than their counterparts with higher cholesterol levels. These individuals also demonstrated an elevated comorbidities rate, such as hypertension and diabetes. Nonetheless, the baseline means for systolic blood pressure and fasting blood glucose were lower in this group. Although the difference was insignificant, the results also revealed that the patients with lower cholesterol levels had a lower smoking percentage than other groups.

Regarding treatment patterns, STEMI was significantly lower among patients with lower cholesterol levels. Conversely, the Killip class and cardiac catheterisation were higher among these patients. Moreover, diuretics and calcium antagonists were more likely to be prescribed to patients with lower cholesterol levels. Nonetheless, they were less likely to receive ACE inhibitors and lipid-lowering agents (other than statins).

### 3.3. Cholesterol and Mortality

Table 3 lists the in-hospital all-cause mortality rates for each TC quartile group evaluated in this study. The cut-off points for each quartile are presented in Table 3. The highest mortality rate, 6.6%, was documented by the Q1 group, followed by Q4 (3.3%), Q2 (3.2%), and Q3 (2.5%). For most patients, CVD was the primary cause of fatality, accounting for 95.8%.

Increased mortality rate was significantly associated with low cholesterol levels, as the patients with the lowest cholesterol levels, those in the Q1 group, had the highest mortality risk. These patients recorded a 2.10 unadjusted OR (95% CI: 1.70 to 2.58), which remained substantial even after adjustments for all significant individual-level variables and multilevel modelling analysis for the cluster or hospital effect. Conclusively, patients with low cholesterol levels had a higher mortality risk even after controlling for potential confounding factors, with a 1.76 adjusted OR and 95% CI of 1.39 to 2.22.

To determine whether the observed association was specific to TC or also evident using a marker of atherogenic lipoprotein burden, non-HDL-C was calculated (TC minus HDL-C) and analysed using the same quartile-based modelling strategy (Appendix A, Table A3). In the fully adjusted multilevel model, patients in the lowest non-HDL-C quartile (Q1) demonstrated a significantly higher risk of in-hospital mortality relative to Q2 (adjusted OR 1.82, 95% CI 1.44–2.31), closely paralleling the magnitude of association observed for TC (adjusted OR 1.76, 95% CI 1.39–2.22). No significant associations were observed for the higher non-HDL-C quartiles (Q3 and Q4) after full adjustment.

### 3.4. Subgroups Analysis of the ACS Classifications

When evaluated according to the ACS stratum (STEMI, NSTEMI, and UA), patients with STEMI and NSTEMI, who documented the lowest cholesterol levels (Q1), had a similar mortality pattern to the overall findings. The NSTEMI patients also exhibited a U-shaped relationship, where those with low and high cholesterol levels exhibited an elevated mortality rate. A different pattern was observed among UA patients, as these individuals did not record a significant correlation between cholesterol level and mortality rate, which was possibly due to the low death rate among this demography.

In NSTEMI, the patients with abnormal or beyond normal range cholesterol levels (Q1, Q3, and Q4) had significantly higher mortality rates compared to their counterparts with normal cholesterol levels (Q2). Comparisons to Q2 also revealed that Q1, Q3, and Q4 had 2.15 (95% CI: 1.15 to 4.02), 2.00 (95% CI: 1.01 to 3.96), and 2.14 (95% CI: 1.08 to 4.24) adjusted OR, respectively. These values suggested that low and high cholesterol levels increased mortality risk in NSTEMI patients. Table A1 in Appendix A summarises the estimates procured during the multilevel analysis performed in this study.

In an additional sex-stratified analysis (Appendix A, Table A4), low admission TC (Q1) remained independently associated with increased in-hospital mortality in both men (adjusted OR 1.58, 95% CI 1.23–2.04) and women (adjusted OR 3.22, 95% CI 1.57–6.63) relative to Q2. Among women, the highest TC quartile (Q4) was also associated with increased in-hospital mortality (adjusted OR 2.42, 95% CI 1.14–5.12), whereas this association was not observed among men. Given the smaller number of female participants in this cohort, these sex-specific findings should be interpreted with caution and warrant confirmation in future studies with larger female subpopulations.

## 4. Discussion

### 4.1. Comparison with Similar Study

Previous studies reported that low levels of cholesterol were correlated with between 1.5% to 8.9% increased mortality rates [9,10,11,12,13,14]. Following multivariate analysis, which involves adjusting for confounding factors, the studies also indicated that elevated cholesterol levels exerted a protective effect on mortality. These findings revealed that, compared to the reference group of low cholesterol levels, the odds or hazard ratios (measures of association) for high cholesterol levels ranged from 0.31 to 0.91. This study shows a similar pattern with previous literature, not only with TC but with other lipid parameters as well. The consistency of this association across TC and non-HDL-C supports the robustness of the low-cholesterol mortality relationship observed in this cohort. Non-HDL-C reflects the total burden of atherogenic lipoproteins and, unlike calculated LDL-C, does not require fasting for accurate measurement [22]. This concordance suggests that the observed mortality signal is not specific to one lipid parameter, but may instead reflect a shared underlying process, such as an acute-phase reduction in circulating lipoproteins, affecting multiple cholesterol fractions. Therefore, higher cholesterol levels might be associated with lower mortality risks.

Nevertheless, most of the studies focused on categorising cholesterol levels into low and high. This classification failed to capture the full complexity of the relationship between cholesterol and mortality and potentially the whole pattern of the relationship. Meanwhile, reports that categorised cholesterol into quartiles or tertiles had a superior capacity to evaluate the relationship between cholesterol levels and mortality rates [12,13,14]. The approach also allowed researchers to better capture the potential U-or J-shaped pattern, which can provide more precise information to facilitate comprehending the association.

Although the association between admission cholesterol levels and adverse outcomes among patients with ACS has been reported previously, most of the existing evidence originates from Western populations, such as the large US-based CRUSADE registry [11], or from East Asian cohorts, such as a large Chinese coronary artery disease cohort study [7]. Comparatively, data from Southeast Asia, particularly from large, multi-ethnic nationwide registries, remain limited. Malaysia represents a unique multi-ethnic population comprising Malay, Chinese, Indian, and indigenous groups, each characterised by distinct genetic predispositions, dietary patterns, socioeconomic backgrounds, and cardiovascular risk profiles. The observation of a similar U-shaped association between admission TC and in-hospital mortality within this heterogeneous population suggests that the prognostic value of admission cholesterol may be broadly consistent across diverse ethnic groups and healthcare settings. Accordingly, the novelty of the present study lies not in identifying a previously undescribed association, but in providing external validation of the cholesterol paradox within a large, nationwide, multi-ethnic Southeast Asian cohort, while additionally accounting for inter-hospital variation through multilevel modelling —an analytical approach not consistently applied in previous reports.

Beyond this population-level contribution, restricting the cohort to first-episode ACS and examining subtype-specific heterogeneity, particularly the pronounced U-shaped association observed among patients with NSTEMI, further distinguish this study from much of the existing cholesterol paradox literature, which has more often analysed heterogeneous or recurrent ACS populations without subtype stratification. These findings offered evidence for the complex relationship between cholesterol levels and mortality rates.

### 4.2. Clarifications

It is important to reconcile the present findings with the well-established evidence that lowering LDL-C reduces the long-term incidence of atherosclerotic cardiovascular events, including ACS [23,24]. This body of evidence, derived largely from randomised controlled trials of statins and other lipid-lowering agents, supports a causal role for LDL-C in the chronic development of atherosclerosis over years of exposure. The present study addresses a distinct clinical question: the prognostic significance of a single cholesterol measurement obtained at the time of acute presentation with ACS, in relation to short-term in-hospital mortality. Accordingly, these findings should not be interpreted as evidence against lipid-lowering therapy or as suggesting that lower cholesterol is intrinsically harmful. Rather, consistent with the broader ‘cholesterol paradox’ literature, low cholesterol measured acutely at ACS presentation more plausibly reflects an underlying state of physiological vulnerability—including systemic inflammation, malnutrition, hepatic dysfunction, or occult chronic illness—that lowers circulating lipoprotein levels as part of the acute-phase response, rather than indicating a genuinely lower atherogenic burden. These two phenomena, the long-term causal role of LDL-C in atherogenesis and the short-term prognostic significance of acutely depressed cholesterol as a marker of illness severity, are not mutually contradictory but instead operate on different timescales and reflect different underlying biology.

Several biological mechanisms may explain the cholesterol paradox observed in the present study. First, low cholesterol may correlate with a pro-inflammatory state rather than a true cardioprotective state. Yang et al. [8] and Nakagomi et al. [25] suggested that low cholesterol levels have been proposed as a biological marker for cancer and chronic illness, thereby increasing the mortality risks of these individuals and affecting their survival rate. Severe chronic illnesses, liver diseases, or cancer could contribute to the elevated mortality rates among individuals with low cholesterol levels. Recent studies have indicated that patients with ACS and low LDL-C levels have a higher inflammatory burden and worse clinical outcomes [26,27]. Consequently, low cholesterol levels may represent a marker of disease severity rather than a direct causal factor for mortality.

Another explanation is that of reverse causality: patients with chronic inflammation, cachexia, malnutrition, liver dysfunction, or occult disease often have lower cholesterol concentrations but at the same time have poorer clinical outcomes [7,28]. Although the present study excluded patients with major chronic illnesses, residual confounding from subclinical disease processes may still exist. A more specific biological mechanism underlying this observation is the hepatic acute-phase response. During acute illness, including the systemic inflammatory response accompanying ACS itself, pro-inflammatory cytokines such as interleukin-6 and tumour necrosis factor-alpha act on the liver to suppress hepatic synthesis and secretion of cholesterol-carrying lipoproteins, while simultaneously altering lipoprotein clearance pathways [29]. This acute-phase-mediated fall in circulating total and LDL-C can occur within hours of an acute inflammatory insult and is independent of any underlying change in atherogenic lipid burden. Consequently, low admission cholesterol measured at the time of ACS presentation may partly reflect the acute severity of the index event itself, rather than solely representing a stable, pre-existing physiological or nutritional state.

Nonetheless, the cholesterol paradox remains evident from the results in this study, even after excluding data of patients with severe chronic illnesses. Almost all mortality (95.8%) of the patients assessed was also due to CVD. Therefore, additional information on liver function or inflammatory markers could provide insights into the role of hepatic dysfunction in the cholesterol paradox. Nevertheless, this study could not assess this hypothesis accurately due to insufficient data.

Cholesterol has important physiological functions in addition to its role in atherosclerosis. It is a basic structural element of cell membranes and has a part in cellular signaling, immune regulation, tissue repair, and host defense mechanisms [30]. Thus, the low cholesterol levels may impair the body’s ability to respond adequately to acute physiological stress, infection, and tissue injury [31].

Wang et al. [7] reported a correlation between poor nutritional status in elderly patients with low LDL-C levels. Inadequate dietary intake among the respondents could affect nutrient absorption and metabolism among the elderly, contributing to the observation. Similarly, the nutritional status of the patients evaluated in this study could explain the cholesterol paradox observed, considering that low cholesterol levels, particularly LDL-C, have been linked to poor nutritional status or malnutrition. Not receiving sufficient nutrients, including essential nutrients, such as vitamins and minerals, can render the body unable to function effectively. This condition is known as malnutrition, which can lead to a myriad of health issues, including weakened immune systems and increased risks of infection. Therefore, malnutrition potentially elevates illness and mortality rates [6].

Low cholesterol levels have been linked to poor health. Consequently, individuals with low cholesterol levels also have an elevated morbidity rate and advancing age, which increases their mortality rate. The results in this study also demonstrated a similar pattern. Patients with low cholesterol levels were older and recorded an increased prevalence of comorbidities. Nago et al. [13] also observed that patients with comorbidities had frequent interactions with the medical field before ACS events. Therefore, the likelihood of elevated cholesterol levels among these individuals decreased due to medical interventions. In Malaysia, individuals with chronic diseases, such as hypertension and diabetes, typically visit a health clinic regularly for health monitoring [32,33]. Appropriate interventions, such as lifestyle modifications or medication therapy, will then be prescribed to patients with health conditions deemed unsatisfactory. This strategy could explain the well-controlled cholesterol levels of the patients, despite having chronic diseases. Comparable patterns and findings have also been observed in other studies [7,9,11]. Overall, comorbidities associated with low cholesterol levels and poor health outcomes can only partially explain the correlation between low cholesterol levels and increased mortality.

Sex-based differences in the presentation and prognosis of ACS are well described, with women more likely to present with atypical symptoms, delayed diagnosis, and under-treatment relative to men [34]. In the present study, low admission TC was independently associated with increased in-hospital mortality in both sexes, whereas elevated cholesterol (Q4) was associated with increased mortality only among women. This pattern may reflect sex-specific differences in lipid metabolism, hormonal influences on lipoprotein profiles, and differential burden of comorbidities at ACS presentation. However, given the relatively smaller number of women in this cohort and the correspondingly wide confidence intervals, this observation should be regarded as hypothesis-generating rather than definitive, and warrants further investigation in adequately powered, sex-balanced cohorts.

It is important to note that admission TC should be interpreted as one informative component of the broader lipid profile, rather than as an isolated determinant of ACS prognosis. Individual lipid fractions are known to play distinct pathophysiological roles: LDL-C promotes atherosclerosis through cholesterol deposition within the arterial wall and progressive plaque formation [35], HDL-cholesterol exerts cardioprotective effects through reverse cholesterol transport, anti-inflammatory, and antioxidant mechanisms [36], and triglyceride-rich lipoproteins, including VLDL, contribute to residual cardiovascular risk through pro-inflammatory and pro-atherogenic pathways distinct from those of LDL-C, including remnant lipoprotein accumulation within the arterial wall and endothelial dysfunction [37]. These differential mechanisms illustrate why the individual lipid fractions cannot be assumed to behave interchangeably, and why admission TC, while informative, captures only part of the overall atherogenic and prognostic picture in ACS. In supplementary analyses (Appendix A, Table A3), lower LDL-C and HDL-C were each independently associated with increased in-hospital mortality, closely paralleling the pattern observed for TC, whereas triglyceride levels showed no significant independent association. This concordance across multiple lipid fractions supports the interpretation that the low-lipid mortality association identified in this study reflects a shared underlying physiological vulnerability rather than an effect specific to TC alone. Future studies incorporating comprehensive lipid fractionation, apolipoproteins, lipoprotein(a) [38], and other emerging biomarkers are warranted to further refine cardiovascular risk stratification in ACS.

### 4.3. U-Shaped Association in NSTEMI

An important finding of the present study was that the U-shaped association between cholesterol levels and mortality was observed primarily among NSTEMI patients. Compared with patients whose cholesterol levels were within the normal range, those with both lower and higher cholesterol levels experienced significantly greater mortality risks.

Several explanations may account for this observation. NSTEMI patients are generally older and have a higher prevalence of cardiovascular risk factors and comorbidities, including diabetes mellitus, hypertension, chronic kidney disease, and metabolic disorders, compared with STEMI patients [27,39]. Consequently, cholesterol levels among NSTEMI patients may reflect not only atherosclerotic burden but also nutritional status, inflammatory activity, and overall physiological reserve. In this context, low cholesterol levels may represent frailty, malnutrition, or chronic systemic illness, whereas elevated cholesterol levels may indicate greater underlying atherosclerotic burden and cardiovascular risk [7,26]. The mechanism behind elevated cholesterol is different from that of low cholesterol. Over time, high LDL-C builds up fatty deposits inside the artery wall, forming plaques with a soft, lipid-rich core covered by a thin, fragile cap. If this cap tears, the plaque’s contents trigger a blood clot, blocking the artery and worsening the heart attack [35]. Unlike the sudden drop in cholesterol seen during acute illness, this is a slow, long-term process that builds up years before the ACS event itself. These mechanisms may contribute simultaneously to the U-shaped mortality pattern observed among NSTEMI patients.

By contrast, STEMI is typically precipitated by acute plaque rupture and complete coronary artery occlusion. Therefore, the severity of the acute ischemic event may exert a stronger influence on short-term mortality than baseline cholesterol levels, potentially attenuating the relationship between cholesterol and mortality in this subgroup [9]. Furthermore, the low mortality rate observed among unstable angina patients may have reduced the statistical power required to detect a significant association between cholesterol levels and mortality outcomes.

These findings suggest that cholesterol levels may have greater prognostic value among NSTEMI patients and could potentially contribute to future risk stratification strategies in this population.

### 4.4. Contribution to Clinical Practice

The present findings have several implications for clinical practice. This study emphasised the importance of routine health monitoring, particularly for individuals between the ages of 30 and 70, for clinical significance. Based on the findings, patients presenting with first-episode ACS and cholesterol levels outside the reference range were more likely to experience in-hospital mortality, with almost double the fatality risk.

This study may have important implications for clinical risk stratification in ACS patients. Current ACS management guidelines are largely focused on hypercholesterolemia as a modifiable cardiovascular risk factor. However, recent evidence suggests that both low and high cholesterol levels may identify patients at increased risk for adverse outcomes. Therefore, cholesterol measurements obtained during hospitalisation may provide prognostic information in addition to traditional cardiovascular risk assessment. Importantly, unexpectedly low admission cholesterol should not be interpreted as a favourable prognostic sign; rather, it may reflect underlying physiological vulnerability, including frailty, malnutrition, systemic inflammation, or occult comorbidity [28,29]. These findings do not contradict the established benefit of LDL-cholesterol lowering for long-term atherosclerotic risk reduction [23,24], but instead offer complementary, admission-stage prognostic information. Specifically, in NSTEMI patients, abnormal cholesterol levels may assist clinicians in identifying patients for more vigilant follow-up and more aggressive management. Cholesterol alone should not be used as a prognostic marker, but lipid measurements may improve early detection of high-risk patients when integrated into existing risk assessment schemes. As ACS is a multifactorial condition, admission cholesterol should be interpreted alongside established risk factors and prognostic indicators—such as Killip class, hypertension, diabetes, and smoking status—rather than in isolation. These observations should be regarded as hypothesis-generating, and prospective validation in contemporary cohorts is required before admission cholesterol could be formally incorporated into risk-stratification tools or treatment decision-making in ACS.

Moreover, the findings underline the need to investigate the baseline nutritional state, frailty, inflammatory burden, and comorbidity profile in ACS patients with unexpectedly low cholesterol levels. Future studies should evaluate whether adding cholesterol measurements to established prognostic factors improves mortality prediction models in ACS populations.

The outcomes in this study are particularly relevant to Malaysia, as approximately half of the adults in the nation, aged 30 to 70, have elevated cholesterol levels [40]. Accordingly, public health authorities should consider increasing public awareness of monitoring health status, especially in populations that are more commonly diagnosed with hypercholesterolemia. Healthcare providers can also foster cholesterol levels and other health indicators monitoring among the public to improve their overall health outcomes, as a strategy to facilitate CVD prevention and management.

### 4.5. Limitations

This study has several limitations that should be considered when interpreting the findings. First, the case-selection process illustrated in Figure 1 warrants further consideration, as it reduced the eligible registry population from 48,851 to a final analytic cohort of 16,860. Several exclusions were made on purpose, to match the study’s aims, rather than being sources of bias. Exclusion of non-initial ACS presentations and duplicate records (*n* = 4376) and of patients outside the pre-specified 30–70-year age range (*n* = 8731) restricted the cohort to first-episode ACS in the age group that best represents ACS patients in Malaysia [18]. Exclusion of patients with pre-existing myocardial infarction (*n* = 3280), documented coronary artery disease (*n* = 2021), heart failure (*n* = 487), or chronic renal or lung disease (*n* = 939) was done to separate the cholesterol–mortality link from the effect of existing heart or major organ disease. As a result, the findings apply specifically to patients aged 30–70 having their first ACS episode without major pre-existing heart or organ disease; they may not extend directly to patients outside this age range, especially older adults, whose other illnesses and frailty could change how cholesterol relates to mortality, or to the broader ACS population as a whole. The exclusion of patients without a lipid profile recorded within 48 h of admission (*n* = 11,025) is the step most likely to have caused selection bias, because patients who did not have their lipids tested may not be a random group. For example, patients who died very soon after arrival, arrived critically ill, or were rushed for urgent treatment may have been less likely to have blood drawn in time. If these sicker patients were left out, the true mortality risk linked to low cholesterol may be even higher than what this study found. Finally, exclusion due to missing outcome data (*n* = 123) was minimal, accounting for less than 0.3% of the pre-exclusion cohort, and is unlikely to have materially influenced the results.

Second, the finding in this study indicated an association between cholesterol levels and mortality. However, this study’s cross-sectional design means it cannot prove that low cholesterol causes higher mortality, only that the two are linked. Cholesterol was also measured only once, at admission, so it is unclear whether low cholesterol was a long-standing state or a temporary change around the time of the ACS event. Therefore, longitudinal studies assessing the causal relationship between the variables, where patients are observed over a period, and their cholesterol levels are measured at multiple points, are vital.

An additional limitation relates to the study period. Although the NCVD-ACS registry continues to enrol patients, the present analysis was restricted to records from January 2006 to December 2015, reflecting the most recent validated dataset available when the study was designed and ethically approved. ACS management has evolved considerably since 2015, including wider use of high-intensity statins, newer antiplatelet agents, and improved access to timely revascularisation [41]. Consequently, the absolute mortality rates observed in this cohort may not fully reflect contemporary clinical outcomes, and the generalisability of these findings to current practice should be interpreted with caution. Future studies validating these findings using more recent NCVD-ACS registry data are therefore warranted.

Assessments that involve large datasets commonly need to face missing data issues, which was also the case for this study. This was addressed using multivariate imputation by chained equations (MICE), a statistical method that estimates plausible values for missing data. Twenty imputed datasets were generated using all variables included in the final analytical model. Parameter estimates from each imputed dataset were combined using Rubin’s rules to obtain pooled estimates and standard errors. Approximately 18.0% of records were missing data for history of dyslipidaemia, a covariate conceptually related to the lipid focus of this study, although distinct from the fully observed primary exposure variable (admission TC). As this variable was used only for adjustment and did not affect who was included in the cohort, it is unlikely to have biased the main cholesterol–mortality association. Nonetheless, this represents a specific source of residual uncertainty that should be considered when interpreting the adjusted estimates. The missing values and imputation methods for each variable are listed in Table A2 in Appendix A.

A further limitation is the absence of genomic and advanced lipid marker data in the NCVD-ACS registry. In recent years, clonal haematopoiesis of indeterminate potential (CHIP) has been identified as an important age-related somatic risk factor for atherosclerotic cardiovascular disease and adverse cardiovascular outcomes, independent of conventional lipid profiles [42]. Similarly, lipoprotein (a) [Lp(a)] is an independent, genetically determined lipid marker increasingly recognised to have a causal role in atherosclerotic cardiovascular disease [38], but is not routinely measured within the registry. As the NCVD-ACS registry captures neither genetic nor genomic information, including CHIP status, nor Lp(a) levels, for patients enrolled at any time point, we were unable to evaluate whether these emerging risk markers contribute to, mediate, or confound the association between admission TC and in-hospital mortality observed in this study. Future research integrating national cardiovascular registries with genomic profiling and standardised Lp(a) measurement is warranted to clarify their potential role in the cholesterol paradox among patients with ACS.

Several other factors could also have influenced the link between cholesterol levels and mortality, but were not available in this study: inflammatory markers (e.g., C-reactive protein), detailed liver and kidney function, validated frailty assessments, socioeconomic status, medication records, dietary patterns, nutrition status, physical activity, and menopausal status. In particular, as endogenous oestrogen is known to influence lipid metabolism and vascular function, menopausal status may partly underlie the sex-related differences observed in this study (Section 3.4, Appendix A, Table A4) [43], though this could not be directly evaluated as the NCVD-ACS registry does not record menopausal status, age at menopause, or hormone replacement therapy use. The registry similarly does not capture structured data on pre-admission lipid-lowering therapy, such as statin use; since statins are known to lower cholesterol pharmacologically [23], their omission represents a further potential source of residual confounding. Body weight, although recorded in the NCVD-ACS registry, was missing in more than 50% of the cohort; consequently, body mass index was not included in the multivariable models, as complete-case analysis risked selection bias while imputing such a high proportion of missing data could compromise the reliability of the estimates, and residual confounding related to adiposity cannot be excluded. As the NCVD-ACS registry was designed primarily to capture cardiovascular-specific risk factors and comorbidities, it does not systematically record non-cardiovascular conditions such as malignancy, autoimmune or collagenous diseases, chronic infectious diseases, thyroid dysfunction, or psychiatric disorders, which are also recognised to influence serum cholesterol levels [44]; consequently, these conditions could not be identified or excluded from the present cohort. Overall, the unavailability of these variables limits how fully this study can explain the cholesterol paradox.

## 5. Conclusions

The in-hospital mortality rates of patients experiencing a first episode of ACS were considerably associated with their cholesterol levels. Both low and high cholesterol levels were associated with a higher risk of in-hospital mortality, revealing a U-shaped pattern, particularly among NSTEMI patients, where a normal cholesterol level was correlated with the lowest risk of outcome events. These findings support the existence of a cholesterol paradox and suggest that cholesterol levels may reflect not only cardiovascular risk but also overall physiological reserve and disease severity. Further prospective studies are required to clarify the biological mechanisms underlying this relationship.

## Figures and Tables

**Table 1:** The Baseline characteristics of patients according to TC quartile.

Characteristic	Q1 *n* = 3940	Q2 *n* = 4488	Q3 *n* = 4214	Q4 *n* = 4218	*p*-Value ^a^	*p*-Value ^b^
Age (years)	55.7 (8.9)	54.4 (8.9)	53.4 (8.8)	52.8 (8.9)	<0.001	<0.001
Gender (Male)	3239 (82.2)	3794 (84.5)	3592 (85.2)	3517 (83.4)	0.001	<0.001
Race					<0.001	<0.001
Malay	1876 (47.6)	2301 (51.3)	2474 (58.7)	2866 (67.9)		
Chinese	925 (23.5)	998 (22.2)	723 (17.2)	550 (13.0)		
Indian	912 (23.1)	959 (21.4)	811 (19.2)	639 (15.1)		
Smoking	2487 (63.1)	2978 (66.4)	2869 (68.1)	2881 (68.3)	<0.001	
Family history of CVD	484 (12.3)	556 (12.4)	584 (13.9)	605 (14.3)	0.008	
Dyslipidaemia	1119 (28.4)	1144 (25.5)	1071 (25.4)	1073 (25.4)	0.003	
Hypertension	2315 (58.8)	2309 (51.4)	1898 (45.0)	1838 (43.6)	<0.001	<0.001
Diabetes	1783 (45.3)	1634 (36.4)	1323 (31.4)	1359 (32.2)	<0.001	<0.001
CVA	143 (3.6)	110 (2.5)	82 (1.9)	79 (1.9)	<0.001	0.004
Peripheral vascular disease	11 (0.3)	9 (0.2)	5 (0.1)	4 (0.1)	0.171	
HR (beats/minute)	83.0 (21.4)	81.8 (20.3)	82.0 (19.7)	83.5 (19.7)	<0.001	0.002
SBP (mmHg)	133.6 (27.8)	136.6 (27.2)	139.2 (27.5)	141.5 (28.7)	<0.001	<0.001
DBP (mmHg)	79.6 (17.5)	82.0 (17.2)	84.0 (17.3)	85.4 (18.0)	<0.001	
FBG (mmol/L)	8.21 (3.97)	8.20 (3.80)	8.19 (3.68)	8.75 (4.26)	<0.001	<0.001
ECG abnormality type						
ST-segment (≥1 mm)	1170 (29.7)	1419 (31.6)	1312 (31.1)	1351 (32.0)	0.118	
ST-segment (≥2 mm)	1318 (33.5)	1737 (38.7)	1735 (41.2)	1909 (45.3)	<0.001	
ST-segment (≥0.5 mm)	799 (20.3)	798 (17.8)	809 (19.2)	792 (18.8)	0.033	
T-wave inversion	763 (19.4)	821 (18.3)	747 (17.7)	671 (15.9)	0.001	
BBB	136 (3.5)	117 (2.6)	118 (2.8)	98 (2.3)	0.016	
ECG abnormality location						
Inferior leads	1541 (39.1)	1782 (39.7)	1607 (38.1)	1536 (36.4)	0.011	
Anterior leads	1623 (41.2)	1990 (44.3)	1989 (47.2)	2254 (53.4)	<0.001	<0.001
Lateral leads	929 (23.6)	1127 (25.1)	1134 (26.9)	1172 (27.8)	<0.001	<0.001
True posterior	226 (5.7)	307 (6.8)	255 (6.1)	239 (5.7)	0.085	
Right ventricle	211 (5.4)	232 (5.2)	214 (5.1)	189 (4.5)	0.291	

Note: Categorical data are presented as frequency (percentage). Numerical data, such as age, heart rate, SBP, DBP, and FBG, were presented as mean (SD), as the distributions were approximately normal, ^a^ univariable analysis: ANOVA test was applied to compare the mean between TC groups, whereas the Chi-squared test was used to compare the proportion, ^b^ multivariable analysis: All significant factors in univariable analysis (*p* < 0.05) were included in the multivariable analysis and further analysis using multilevel modelling. Only significant *p*-values were presented. TC: total cholesterol, HR: heart rate, SBP: systolic blood pressure, DBP: diastolic blood pressure, FBG: fasting blood glucose, CVA: cerebrovascular accident, ECG: electrocardiogram, BBB: bundle branch block, and SD: standard deviation.

**Table 2:** The treatment patterns of patients according to TC quartile.

Characteristic	Q1 *n* = 3940	Q2 *n* = 4488	Q3 *n* = 4214	Q4 *n* = 4218	*p*-Value ^a^	*p*-Value ^b^
ACS Stratum					<0.001	<0.001
STEMI	2283 (57.9)	2887 (64.3)	2834 (67.3)	3003 (71.2)		
NSTEMI	863 (21.9)	890 (19.8)	777 (18.4)	761 (18.0)		
UA	794 (20.2)	711 (15.8)	603 (14.3)	454 (10.8)		
Killip class (III & IV)	574 (14.6)	485 (10.8)	377 (8.9)	443 (10.5)	<0.001	<0.001
Cardiac catheterisation	1505 (38.2)	1676 (37.3)	1520 (36.1)	1436 (34.0)	0.001	<0.001
PCI	1106 (28.1)	1286 (28.7)	1167 (27.7)	1106 (26.2)	0.075	
CABG	46 (1.2)	54 (1.2)	43 (1.0)	36 (0.9)	0.379	
Aspirin	3779 (95.9)	4340 (96.7)	4040 (95.9)	4081 (96.8)	0.040	
ADP antagonist	3345 (84.9)	3821 (85.1)	3531 (83.8)	3572 (84.7)	0.335	
GP receptor inhibitor	101 (2.6)	100 (2.2)	91 (2.2)	103 (2.4)	0.594	
Unfractionated heparin	419 (10.6)	523 (11.7)	506 (12.0)	498 (11.8)	0.220	
LMWH	1268 (32.2)	1513 (33.7)	1338 (31.8)	1315 (31.2)	0.069	
Beta-blocker	2341 (59.4)	2749 (61.3)	2691 (63.9)	2737 (64.9)	<0.001	
ACE inhibitor	1953 (49.6)	2356 (52.5)	2367 (56.2)	2388 (56.6)	<0.001	<0.001
ARB	171 (4.3)	184 (4.1)	134 (3.2)	122 (2.9)	0.001	
Statin	3595 (91.2)	4140 (92.2)	3885 (92.2)	3920 (92.9)	0.043	
Other lipid-lowering agents	105 (2.7)	125 (2.8)	133 (3.2)	155 (3.7)	0.034	0.036
Diuretic	748 (19.0)	755 (16.8)	700 (16.6)	876 (20.8)	<0.001	<0.001
Calcium antagonist	362 (9.2)	349 (7.8)	265 (6.3)	295 (7.0)	<0.001	0.017
Oral hypoglycaemic agent	1008 (25.6)	974 (21.7)	866 (20.6)	870 (20.6)	<0.001	
Insulin	951 (24.1)	921 (20.5)	787 (18.7)	911 (21.6)	<0.001	
Anti-arrhythmic agent	217 (5.5)	208 (4.6)	193 (4.6)	177 (4.2)	0.041	

Note: Categorical data are presented as frequency (percentage), ^a^ univariable analysis: Chi-squared test compared the proportion between TC groups, ^b^ multivariable analysis: All significant factors in univariable analysis (*p* < 0.05) were included in the multivariable analysis, and further analysis with multilevel modelling, and only significant *p*-values are presented. TC: total cholesterol, ADP: adenosine diphosphate, GP: glycoprotein, ACE: angiotensin-converting enzyme, ARB: angiotensin receptor blocker, and LMWH: low molecular weight heparin.

**Table 3:** The associations between TC level and mortality.

TC Group	TC (mmol/L)	Total	Death (%)	Model 1 * OR (95% CI)	Model 2 ** OR (95% CI)	Model 3 *** OR (95% CI)	Model 4 **** OR (95% CI)
Overall (*n* = 16,860)							
Q1 (1st–25th)	2.00–4.49	3940	258 (6.6)	2.10 (1.70 to 2.58) ^	1.76 (1.41 to 2.20) ^	1.76 (1.39 to 2.22) ^	1.76 (1.39 to 2.22) ^
Q2 (26th–50th)	4.50–5.36	4488	145 (3.2)	1.00 (Ref)	1.00 (Ref)	1.00 (Ref)	1.00 (Ref)
Q3 (51st–75th)	5.37–6.25	4214	107 (2.5)	0.78 (0.61 to 1.01)	0.85 (0.65 to 1.10)	0.90 (0.69 to 1.19)	0.90 (0.68 to 1.19)
Q4 (76th–100th)	6.26–16.6	4218	138 (3.3)	1.01 (0.80 to 1.28)	1.02 (0.80 to 1.32)	1.03 (0.79 to 1.33)	1.03 (0.79 to 1.34)
STEMI (*n* = 11,007)							
Q1 (1st–25th)	2.00–4.49	2283	207 (9.1)	2.20 (1.75 to 2.77) ^	1.80 (1.41 to 2.30) ^	1.72 (1.33 to 2.23) ^	1.72 (1.33 to 2.24) ^
Q2 (26th–50th)	4.50–5.36	2887	125 (4.3)	1.00 (Ref)	1.00 (Ref)	1.00 (Ref)	1.00 (Ref)
Q3 (51st–75th)	5.37–6.25	2834	78 (2.8)	0.63 (0.47 to 0.83) ^	0.67 (0.49 to 0.90) ^	0.73 (0.53 to 1.01)	0.73 (0.53 to 1.01)
Q4 (76th–100th)	6.26–16.6	3003	112 (3.7)	0.86 (0.66 to 1.11)	0.86 (0.65 to 1.14)	0.86 (0.65 to 1.16)	0.85 (0.64 to 1.15)
NSTEMI (*n* = 3291)							
Q1 (1st–25th)	2.00–4.49	863	46 (5.3)	3.08 (1.73 to 5.48) ^	2.54 (1.40 to 4.62) ^	2.49 (1.35 to 4.60) ^	2.15 (1.15 to 4.02) ^
Q2 (26th–50th)	4.50–5.36	890	16 (1.8)	1.00 (Ref)	1.00 (Ref)	1.00 (Ref)	1.00 (Ref)
Q3 (51st–75th)	5.37–6.25	777	25 (3.2)	1.82 (0.96 to 3.43)	2.06 (1.07 to 3.97) ^	2.04 (1.04 to 4.01) ^	2.00 (1.01 to 3.96) ^
Q4 (76th–100th)	6.26–16.6	761	24 (3.2)	1.78 (0.94 to 3.37)	2.12 (1.09 to 4.11) ^	2.25 (1.14 to 4.43) ^	2.14 (1.08 to 4.24) ^
UA (*n* = 2562)							
Q1 (1st–25th)	2.00–4.49	794	5 (0.6)	1.12 (0.30 to 4.19)	1.11 (0.28 to 4.45)	0.86 (0.20 to 3.61)	0.99 (0.55 to 1.77)
Q2 (26th–50th)	4.50–5.36	711	4 (0.6)	1.00 (Ref)	1.00 (Ref)	1.00 (Ref)	1.00 (Ref)
Q3 (51st–75th)	5.37–6.25	603	4 (0.7)	1.18 (0.29 to 4.74)	1.60 (0.37 to 6.94)	1.65 (0.37 to 7.37)	1.04 (0.56 to 1.94)
Q4 (76th–100th)	6.26–16.6	454	2 (0.4)	0.78 (0.14 to 4.29)	1.06 (0.18 to 6.37)	0.97 (0.16 to 6.03)	1.01 (0.51 to 2.01)

Note: TC: total cholesterol, OR: odds ratio, CI: confidence interval. The percentage was based on the number of deaths/number of total cases × 100%, ^ the ratio was significant (*p* < 0.05). * Model 1 was unadjusted (no confounders in the model), ** Model 2 was adjusted for baseline characteristics only (age, gender, race, smoking status, hypertension, diabetes, CVA, HR, SBP, FBG, anterior ECG, and lateral ECG), *** Model 3 was adjusted for baseline characteristics and treatment patterns, and **** Model 4 was adjusted using multilevel modeling for baseline characteristics, treatment patterns, and SDP-level characteristics.

## Data Availability

The data that support the findings of this study are available from the NCVD-ACS Registry. Data are available upon reasonable request and with permission from the National Heart Association of Malaysia (secretariat@malaysianheart.org), subject to data release procedures and legal agreements.

## Appendix A

**Table A1:** The multilevel logistic regression models demonstrating the associations of TC with mortality according to the ACS stratum adjusted to possible confounding factor.

Characteristic	Overall *n* = 16,860	STEMI *n* = 11,007	NSTEMI *n* = 3291
Individual-level attribute			
TC Group			
Q2	1.00 (Ref)	1.00 (Ref)	1.00 (Ref)
Q1	1.76 (1.39 to 2.22)	1.72 (1.33 to 2.24)	2.15 (1.15 to 4.02)
Q3	0.90 (0.68 to 1.19)	0.73 (0.53 to 1.01)	2.00 (1.01 to 3.96)
Q4	1.03 (0.79 to 1.34)	0.85 (0.64 to 1.15)	2.14 (1.08 to 4.24)
Age (years)	1.04 (1.03 to 1.06)	1.05 (1.03 to 1.06)	1.03 (1.00 to 1.06)
Gender (male)	1.24 (0.93 to 1.64)	1.06 (0.76 to 1.47)	2.11 (1.11 to 4.01)
Race			
Malay	0.90 (0.55 to 1.47)	0.95 (0.55 to 1.65)	0.54 (0.20 to 1.47)
Chinese	0.88 (0.53 to 1.47)	0.97 (0.55 to 1.74)	0.46 (0.16 to 1.31)
Indian	0.72 (0.42 to 1.23)	0.75 (0.40 to 1.38)	0.45 (0.15 to 1.39)
Others	1.00 (Ref)	1.00 (Ref)	1.00 (Ref)
Smoking	0.87 (0.70 to 1.08)	0.84 (0.65 to 1.09)	0.84 (0.50 to 1.39)
Hypertension	1.56 (1.28 to 1.90)	1.43 (1.15 to 1.78)	1.87 (1.14 to 3.06)
Diabetes	0.86 (0.70 to 1.06)	0.94 (0.75 to 1.19)	0.74 (0.45 to 1.22)
CVA	1.39 (0.86 to 2.24)	1.43 (0.83 to 2.49)	0.89 (0.25 to 3.19)
HR (beats/min)	1.02 (1.01 to 1.02)	1.01 (1.01 to 1.02)	1.02 (1.01 to 1.02)
SBP (mmHg)	0.98 (0.98 to 0.99)	0.99 (0.98 to 0.99)	0.98 (0.97 to 0.99)
FBG (mmol/L)	1.10 (1.08 to 1.12)	1.11 (1.09 to 1.13)	1.08 (1.04 to 1.13)
Anterior leads	1.06 (0.88 to 1.28)	1.10 (0.89 to 1.37)	1.02 (0.64 to 1.62)
Lateral leads	1.36 (1.11 to 1.66)	1.54 (1.22 to 1.94)	1.05 (0.66 to 1.66)
ACS Stratum			
STEMI	5.05 (2.94 to 8.67)		
NSTEMI	3.87 (2.21 to 6.79)		
UA	1.00 (Ref)		
Killip Class (III and IV)	4.03 (3.34 to 4.88)	4.06 (3.28 to 5.03)	3.97 (2.46 to 6.41)
Cardiac catheterisation	0.54 (0.44 to 0.67)	0.53 (0.42 to 0.67)	0.45 (0.26 to 0.76)
ACE inhibitor	0.33 (0.27 to 0.41)	0.29 (0.23 to 0.37)	0.44 (0.28 to 0.71)
Other lipid-lowering agents	0.64 (0.32 to 1.27)	0.76 (0.36 to 1.60)	0.34 (0.04 to 2.59)
Diuretic	1.72 (1.42 to 2.09)	1.87 (1.50 to 2.33)	1.23 (0.76 to 1.99)
Calcium antagonist	0.77 (0.50 to 1.19)	0.84 (0.48 to 1.48)	0.70 (0.31 to 1.56)
SDP-level attribute			
Target population (′00,000)	1.03 (0.97 to 1.11)	1.02 (0.96 to 1.09)	1.09 (0.94 to 1.26)
Number of cardiologists	0.99 (0.97 to 1.01)	0.99 (0.96 to 1.01)	1.01 (0.96 to 1.05)
Number of cath labs	1.01 (0.89 to 1.16)	1.04 (0.91 to 1.18)	0.92 (0.70 to 1.20)
Random effects			
Estimate (SE)	0.106 (0.060)	0.091 (0.060)	0.299 (0.240)
Model fit			
−2LL	123,000	76,640	23,101
AIC	123,001	76,642	23,103
BIC	123,009	76,650	23,109

Note: Data are present as OR with 95% CI. TC: total cholesterol, CVA: cerebrovascular accident, HR: heart rate, SBP: systolic blood pressure, FBG: fasting blood glucose, SDP: source data provider, OR: odds ratio, CI: confidence interval, SE: standard error, −2LL: −2 log-likelihood, AIC: Akaike information criterion, and BIC: Bayesian information criterion.

**Table A2:** A summary of missing data and imputation method.

Study Variables	Variable Type	Percentage Missing (%)	Imputation Method
Demographics			
Age at admission (years)	Continuous	0.0	No missingness (predictor only)
Gender	Binary	0.0	No missingness (predictor only)
Race	Categorical	0.0	No missingness (predictor only)
CVD risk factors and comorbidities			
Smoking status	Categorical	3.4	Polytomous regression
Family history of premature CVD	Binary	15.6	Default imputation
Dyslipidemia	Binary	18.0	Default imputation
Hypertension	Binary	11.1	Default imputation
Diabetes	Binary	12.3	Default imputation
Cerebrovascular disease	Binary	13.8	Default imputation
Peripheral vascular disease	Binary	14.5	Default imputation
Baseline presentation			
Heart rate at presentation (beats/min)	Continuous	2.5	Predictive mean matching
Systolic blood pressure (mmHg)	Continuous	2.1	Predictive mean matching
Diastolic blood pressure (mmHg)	Continuous	3.0	Predictive mean matching
Fasting blood glucose (mmol/L)	Continuous	8.6	Predictive mean matching
TC (mmol/L)	Continuous	0.0	No missingness (predictor only)
LDL-C (mmol/L)	Continuous	3.5	Predictive mean matching
HDL-C (mmol/L)	Continuous	3.4	Predictive mean matching
Triglyceride (mmol/L)	Continuous	6.4	Predictive mean matching
Clinical diagnosis and treatment			
ACS Stratum	Categorical	0.0	No missingness (predictor only)
Killip class	Categorical	19.0	Polytomous regression
Cardiac catheterisation	Binary	4.4	Binary logistic regression
Percutaneous coronary intervention	Binary	5.8	Binary logistic regression
Coronary artery bypass	Binary	6.3	Binary logistic regression
Pharmacological therapy			
Aspirin	Binary	1.6	Default imputation
Adenosine-diphosphate (ADP) antagonist	Binary	4.3	Default imputation
GP receptor inhibitor	Binary	13.3	Default imputation
Unfractionated heparin	Binary	12.9	Default imputation
Low-molecular-weight heparin	Binary	11.4	Default imputation
Beta-blocker	Binary	7.6	Default imputation
ACE inhibitor	Binary	8.4	Default imputation
Angiotensin II receptor blocker	Binary	13.4	Default imputation
Statin	Binary	2.3	Default imputation
Other lipid-lowering agents	Binary	13.0	Default imputation
Diuretic	Binary	11.4	Default imputation
Calcium antagonist	Binary	13.0	Default imputation
Oral hypoglycaemic agent	Binary	11.3	Default imputation
Insulin	Binary	11.1	Default imputation
Anti-arrhythmic agent	Binary	13.4	Default imputation

**Table A3:** The associations between other cholesterols level and mortality.

Cholesterols	Level (mmol/L)	Total	Death (%)	Model 1 * OR (95% CI)	Model 2 ** OR (95% CI)	Model 3 *** OR (95% CI)	Model 4 **** OR (95% CI)
LDL cholesterol							
Q1 (1st–25th)	0.50–2.69	4006	249 (6.2)	1.99 (1.61–2.46) ^	1.73 (1.38–2.17) ^	1.75 (1.39–2.22) ^	1.74 (1.37–2.21) ^
Q2 (26th–50th)	2.70–3.49	4375	141 (3.2)	1.00 (Ref)	1.00 (Ref)	1.00 (Ref)	1.00 (Ref)
Q3 (51st–75th)	3.50–4.29	4189	127 (3.0)	0.94 (0.74–1.20)	1.06 (0.82–1.37)	1.14 (0.88–1.49)	1.15 (0.88–1.50)
Q4 (76th–100th)	4.30–14.8	4290	131 (3.1)	0.95 (0.74–1.20)	1.00 (0.77–1.28)	0.98 (0.75–1.28)	1.00 (0.76–1.31)
HDL cholesterol							
Q1 (1st–25th)	0.50–0.89	4051	216 (5.3)	1.71 (1.38–2.13) ^	1.58 (1.26–2.00) ^	1.63 (1.28–2.07) ^	1.59 (1.24–2.03) ^
Q2 (26th–50th)	0.90–1.01	4304	137 (3.2)	1.00 (Ref)	1.00 (Ref)	1.00 (Ref)	1.00 (Ref)
Q3 (51st–75th)	1.02–1.19	3338	109 (3.3)	1.03 (0.80–1.33)	1.00 (0.76–1.31)	1.02 (0.77–1.35)	0.93 (0.70–1.23)
Q4 (76th–100th)	1.20–4.90	5167	186 (3.6)	1.14 (0.91–1.42)	0.98 (0.77–1.24)	0.94 (0.73–1.21)	0.89 (0.69–1.14)
Triglycerides							
Q1 (1st–25th)	0.50–1.19	4019	186 (4.6)	1.17 (0.95–1.44)	1.12 (0.90–1.40)	1.04 (0.83–1.32)	1.04 (0.82–1.31)
Q2 (26th–50th)	1.20–1.57	4393	175 (4.0)	1.00 (Ref)	1.00 (Ref)	1.00 (Ref)	1.00 (Ref)
Q3 (51st–75th)	1.58–2.14	4224	159 (3.8)	0.94 (0.76–1.17)	1.00 (0.80–1.26)	1.03 (0.81–1.31)	1.04 (0.81–1.33)
Q4 (76th–100th)	2.15–15.0	4224	128 (3.0)	0.75 (0.60–0.95) ^	0.78 (0.61–1.01)	0.85 (0.66–1.11)	0.84 (0.65–1.10)
Non-HDL cholesterol							
Q1 (1st–25th)	0.50–3.48	4238	268 (6.3)	2.16 (1.75–2.67) ^	1.85 (1.48–2.31) ^	1.85 (1.46–2.34) ^	1.82 (1.44–2.31) ^
Q2 (26th–50th)	3.49–4.30	4423	134 (3.0)	1.00 (Ref)	1.00 (Ref)	1.00 (Ref)	1.00 (Ref)
Q3 (51st–75th)	4.31–5.19	4115	123 (3.0)	0.99 (0.77–1.26)	1.05 (0.81–1.36)	1.14 (0.87–1.49)	1.13 (0.86–1.49)
Q4 (76th–100th)	5.20–15.6	4076	123 (3.0)	1.00 (0.78–1.28)	1.04 (0.80–1.36)	1.05 (0.80–1.38)	1.04 (0.79–1.37)

Note: OR: odds ratio, CI: confidence interval. The percentage was based on the number of deaths/number of total cases × 100%, ^ the ratio was significant (*p* < 0.05). * Model 1 was unadjusted (no confounders in the model), ** Model 2 was adjusted for baseline characteristics only (age, gender, race, smoking status, hypertension, diabetes, CVA, HR, SBP, FBG, anterior ECG, and lateral ECG), *** Model 3 was adjusted for baseline characteristics and treatment patterns, and **** Model 4 was adjusted using multilevel modeling for baseline characteristics, treatment patterns, and SDP-level characteristics.

**Table A4:** The associations between TC level and mortality by gender.

Gender	Level (mmol/L)	Total	Death (%)	Model 1 * OR (95% CI)	Model 2 ** OR (95% CI)	Model 3 *** OR (95% CI)	Model 4 **** OR (95% CI)
Male (*n* = 14,142)							
Q1 (1st–25th)	2.00–4.49	3239	212 (6.5)	1.97 (1.58–2.47) ^	1.60 (1.26 to 2.03) ^	1.58 (1.23 to 2.03) ^	1.58 (1.23 to 2.04) ^
Q2 (26th–50th)	4.50–5.36	3794	130 (3.4)	1.00 (Ref)	1.00 (Ref)	1.00 (Ref)	1.00 (Ref)
Q3 (51st–75th)	5.37–6.25	3592	88 (2.4)	0.71 (0.54–0.93) ^	0.75 (0.56 to 0.99) ^	0.82 (0.61 to 1.10)	0.82 (0.61 to 1.11)
Q4 (76th–100th)	6.26–16.6	3517	106 (3.0)	0.88 (0.67–1.14)	0.88 (0.66 to 1.16)	0.86 (0.65 to 1.15)	0.85 (0.64 to 1.14)
Female (*n* = 2718)							
Q1 (1st–25th)	2.00–4.49	701	46 (6.6)	3.18 (1.76–5.75) ^	2.89 (1.54–5.42) ^	3.17 (1.62–6.20) ^	3.22 (1.57–6.63) ^
Q2 (26th–50th)	4.50–5.36	694	15 (2.2)	1.00 (Ref)	1.00 (Ref)	1.00 (Ref)	1.00 (Ref)
Q3 (51st–75th)	5.37–6.25	622	19 (3.1)	1.43 (0.72–2.83)	1.47 (0.72–3.03)	1.50 (0.70–3.21)	1.51 (0.66–3.44)
Q4 (76th–100th)	6.26–16.6	701	32 (4.6)	2.17 (1.16–4.04) ^	2.10 (1.09–4.08) ^	2.34 (1.17–4.71) ^	2.42 (1.14–5.12) ^

Note: OR: odds ratio, CI: confidence interval. The percentage was based on the number of deaths/number of total cases × 100%, ^ the ratio was significant (*p* < 0.05). * Model 1 was unadjusted (no confounders in the model), ** Model 2 was adjusted for baseline characteristics only (age, gender, race, smoking status, hypertension, diabetes, CVA, HR, SBP, FBG, anterior ECG, and lateral ECG), *** Model 3 was adjusted for baseline characteristics and treatment patterns, and **** Model 4 was adjusted using multilevel modeling for baseline characteristics, treatment patterns, and SDP-level characteristics.

## References

[1] World Health Organization (2020). Global Health Estimates 2020: Deaths by Cause, Age, Sex, by Country and by Region, 2000–2019.

[2] Kucia A.M., Hartley A. (2022). Risk Factors for Cardiovascular Disease. Cardiac Care: A Practical Guide for Nurses.

[3] Díez-Villanueva P., Jiménez-Méndez C., Bonanad C., García-Blas S., Pérez-Rivera Á., Allo G., García-Pardo H., Formiga F., Camafort M., Martínez-Sellés M. (2022). Risk Factors and Cardiovascular Disease in the Elderly. Rev. Cardiovasc. Med..

[4] D’Agostino R.B., Vasan R.S., Pencina M.J., Wolf P.A., Cobain M., Massaro J.M., Kannel W.B. (2008). General Cardiovascular Risk Profile for Use in Primary Care: The Framingham Heart Study. Circulation.

[5] Giugliano D., Longo M., Maiorino M.I., Bellastella G., Chiodini P., Solerte S.B., Esposito K. (2020). Efficacy of SGLT-2 Inhibitors in Older Adults with Diabetes: Systematic Review with Meta-Analysis of Cardiovascular Outcome Trials. Diabetes Res. Clin. Pract..

[6] Cereda E., Pusani C., Limonta D., Vanotti A. (2008). The Association of Geriatric Nutritional Risk Index and Total Lymphocyte Count with Short-Term Nutrition-Related Complications in Institutionalised Elderly. J. Am. Coll. Nutr..

[7] Wang B., Liu J., Chen S., Ying M., Chen G., Liu L., Lun Z., Li H., Huang H., Li Q. (2021). Malnutrition Affects Cholesterol Paradox in Coronary Artery Disease: A 41,229 Chinese Cohort Study. Lipids Health Dis..

[8] Yang W., Bai Y., Xiong Y., Zhang J., Chen S., Zheng X., Meng X., Li L., Wang J., Xu C. (2016). Potentiating the Antitumour Response of CD8+ T Cells by Modulating Cholesterol Metabolism. Nature.

[9] Cheng K.-H., Chu C.-S., Lin T.-H., Lee K.-T., Sheu S.-H., Lai W.-T. (2015). Lipid Paradox in Acute Myocardial Infarction—The Association with 30-Day In-Hospital Mortality. Crit. Care Med..

[10] Ueshima D., Yoshikawa S., Sasaoka T., Hatano Y., Kurihara K., Maejima Y., Isobe M., Ashikaga T. (2019). The Hypercholesterolemia Paradox in Percutaneous Coronary Intervention: An Analysis of a Multicenter PCI Registry. Intern. Med..

[11] Wang T.Y., Newby L.K., Chen A.Y., Mulgund J., Roe M.T., Sonel A.F., Bhatt D.L., DeLong E.R., Ohman E.M., Gibler W.B. (2009). Hypercholesterolemia Paradox in Relation to Mortality in Acute Coronary Syndrome. Clin. Cardiol..

[12] Martin S.S., Faridi K.F., Joshi P.H., Blaha M.J., Kulkarni K.R., Khokhar A.A., Maddox T.M., Havranek E.P., Toth P.P., Tang F. (2015). Remnant Lipoprotein Cholesterol and Mortality after Acute Myocardial Infarction: Further Evidence for a Hypercholesterolemia Paradox from the TRIUMPH Registry. Clin. Cardiol..

[13] Nago N., Ishikawa S., Goto T., Kayaba K. (2011). Low Cholesterol Is Associated with Mortality from Stroke, Heart Disease, and Cancer: The Jichi Medical School Cohort Study. J. Epidemiol..

[14] Xia T.-L., Li Y.-M., Huang F.-Y., Chai H., Huang B.-T., Li Q., Zhao Z.-G., Liao Y.-B., Zou Z.-L., Peng Y. (2019). The Triglyceride Paradox in the Mortality of Coronary Artery Disease. Lipids Health Dis..

[15] Chin S., Jeyaindran S., Azhari R., Wan Azman W., Omar I., Robaayah Z., Sim K. (2008). Acute Coronary Syndrome (ACS) Registry—Leading the Charge for National Cardiovascular Disease (NCVD) Database. Med. J. Malays..

[16] Ahmad W.A.W., Zambahari R., Ismail O., Sinnadurai J., Rosman A., Piaw C.S., Abidin I.Z., Kui-Hian S. (2011). Malaysian National Cardiovascular Disease Database (NCVD)—Acute Coronary Syndrome (ACS) Registry: How Are We Different?. CVD Prev. Control.

[17] Bujang M.A., Tg Abu Bakar Sidik T.M.I., Abdul Ghani P. (2025). Enhancement of systematic sampling with incorporation from consecutive sampling: Systematic sampling with consecutive approach. Thail. Stat..

[18] Ahmad W., Sim K. (2017). Annual Report of the NCVD-ACS Registry, 2014–2015.

[19] Fedder D.O., Koro C.E., L’Italien G.J. (2002). New National Cholesterol Education Program III Guidelines for Primary Prevention Lipid-Lowering Drug Therapy: Projected Impact on the Size, Sex, and Age Distribution of the Treatment-Eligible Population. Circulation.

[20] Mathieu J.E., Taylor S.R. (2007). A Framework for Testing Meso-Mediational Relationships in Organizational Behavior. J. Organ. Behav..

[21] Heck R.H., Thomas S.L. (2020). An Introduction to Multilevel Modeling Techniques: MLM and SEM Approaches.

[22] Mach F., Baigent C., Catapano A.L., Koskinas K.C., Casula M., Badimon L., Chapman M.J., De Backer G.G., Delgado V., Ference B.A. (2020). 2019 ESC/EAS Guidelines for the Management of Dyslipidaemias: Lipid Modification to Reduce Cardiovascular Risk. Eur. Heart J..

[23] Baigent C., Blackwell L., Emberson J., Holland L.E., Reith C., Bhala N., Peto R., Barnes E.H., Keech A., Cholesterol Treatment Trialists’ (CTT) Collaboration (2010). Efficacy and Safety of More Intensive Lowering of LDL Cholesterol: A Meta-Analysis of Data from 170,000 Participants in 26 Randomised Trials. Lancet.

[24] Sabatine M.S., Giugliano R.P., Keech A.C., Honarpour N., Wiviott S.D., Murphy S.A., Kuder J.F., Wang H., Liu T., Wasserman S.M. (2017). Evolocumab and Clinical Outcomes in Patients with Cardiovascular Disease. N. Engl. J. Med..

[25] Nakagomi A., Seino Y., Noma S., Kohashi K., Kosugi M., Kato K., Kusama Y., Atarashi H., Shimizu W. (2014). Relationships between the Serum Cholesterol Levels, Production of Monocyte Proinflammatory Cytokines and Long-Term Prognosis in Patients with Chronic Heart Failure. Intern. Med..

[26] Lu Y., Sun Y., Miao Y., Liu Z., Shen L., He B. (2025). Initial Low-Density Lipoprotein Cholesterol and Inflammation Status Predicts Long-Term Mortality in Patients with Acute Coronary Syndrome in the Chinese Population. Biomedicines.

[27] Jurin I., Jurišić A., Rudež I., Kurtić E., Skorić I., Čikara T., Šipić T., Rudan D., Manola Š., Hadžibegović I. (2024). Outcomes of Patients with Normal LDL-Cholesterol at Admission for Acute Coronary Syndromes: Lower Is Not Always Better. J. Cardiovasc. Dev. Dis..

[28] Czapla M., Juárez-Vela R., Łokieć K., Karniej P. (2021). The Association between Nutritional Status and In-Hospital Mortality Among Patients with Heart Failure-A Result of the Retrospective Nutritional Status Heart Study 2 (NSHS2). Nutrients.

[29] Khovidhunkit W., Kim M.-S., Memon R.A., Shigenaga J.K., Moser A.H., Feingold K.R., Grunfeld C. (2004). Effects of Infection and Inflammation on Lipid and Lipoprotein Metabolism: Mechanisms and Consequences to the Host. J. Lipid Res..

[30] Khalil A. (2024). Nutrition, Lipoproteins and Cardiovascular Diseases. Nutrients.

[31] Bowden R.G., Wilson R.L., Deike E., Gentile M. (2025). Cholesterol Paradox: Understanding and Implications for Clinical Practice and Education. Sci. Arch. Cardiovasc. Med..

[32] Malaysian Endocrine and Metabolic Society, Ministry of Health Malaysia (2020). Clinical Practice Guidelines: Management of Type 2 Diabetes Mellitus.

[33] Malaysian Society of Hypertension, Ministry of Health Malaysia (2018). Clinical Practice Guidelines: Management of Hypertension.

[34] Mehta L.S., Beckie T.M., DeVon H.A., Grines C.L., Krumholz H.M., Johnson M.N., Lindley K.J., Vaccarino V., Wang T.Y., Watson K.E. (2016). Acute Myocardial Infarction in Women: A Scientific Statement from the American Heart Association. Circulation.

[35] Libby P. (2013). Mechanisms of Acute Coronary Syndromes and Their Implications for Therapy. N. Engl. J. Med..

[36] Navab M., Reddy S.T., Van Lenten B.J., Fogelman A.M. (2011). HDL and Cardiovascular Disease: Atherogenic and Atheroprotective Mechanisms. Nat. Rev. Cardiol..

[37] Ginsberg H.N., Packard C.J., Chapman M.J., Borén J., Aguilar-Salinas C.A., Averna M., Ference B.A., Gaudet D., Hegele R.A., Kersten S. (2021). Triglyceride-Rich Lipoproteins and Their Remnants: Metabolic Insights, Role in Atherosclerotic Cardiovascular Disease, and Emerging Therapeutic Strategies—A Consensus Statement from the European Atherosclerosis Society. Eur. Heart J..

[38] Kronenberg F., Mora S., Stroes E.S.G., Ference B.A., Arsenault B.J., Berglund L., Dweck M.R., Koschinsky M., Lambert G., Mach F. (2022). Lipoprotein(a) in Atherosclerotic Cardiovascular Disease and Aortic Stenosis: A European Atherosclerosis Society Consensus Statement. Eur. Heart J..

[39] Cohen-Hagai K., Benchetrit S., Wand O., Grupper A., Shashar M., Solo O., Pereg D., Zitman-Gal T., Haskiah F., Erez D. (2023). The Clinical Significance of LDL-Cholesterol on the Outcomes of Hemodialysis Patients with Acute Coronary Syndrome. Medicina.

[40] Institute for Public Health (IPH) (2020). National Health and Morbidity Survey (NHMS) 2019: Non-Communicable Diseases, Healthcare Demand, and Health Literacy—Key Findings.

[41] Shiyovich A., Shlomo N., Cohen T., Iakobishvili Z., Kornowski R., Eisen A. (2020). Temporal Trends of Patients with Acute Coronary Syndrome and Multi-Vessel Coronary Artery Disease—From the ACSIS Registry. Int. J. Cardiol..

[42] Jaiswal S., Natarajan P., Silver A.J., Gibson C.J., Bick A.G., Shvartz E., McConkey M., Gupta N., Gabriel S., Ardissino D. (2017). Clonal Hematopoiesis and Risk of Atherosclerotic Cardiovascular Disease. N. Engl. J. Med..

[43] El Khoudary S.R., Aggarwal B., Beckie T.M., Hodis H.N., Johnson A.E., Langer R.D., Limacher M.C., Manson J.E., Stefanick M.L., Allison M.A. (2020). Menopause Transition and Cardiovascular Disease Risk: Implications for Timing of Early Prevention: A Scientific Statement from the American Heart Association. Circulation.

[44] Vodnala D., Rubenfire M., Brook R.D. (2012). Secondary Causes of Dyslipidemia. Am. J. Cardiol..

